# The Role of Ferromagnetic Layer Thickness and Substrate Material in Spintronic Emitters

**DOI:** 10.3390/nano13111710

**Published:** 2023-05-23

**Authors:** Arseniy Buryakov, Pavel Avdeev, Dinar Khusyainov, Nikita Bezvikonnyy, Andreas Coclet, Alexey Klimov, Nicolas Tiercelin, Sergey Lavrov, Vladimir Preobrazhensky

**Affiliations:** 1 Department of Nanoelectronics, MIREA—Russian Technological University, 78 Vernadsky Avenue, 119454 Moscow, Russia; avdeev_p@mirea.ru (P.A.); bezvikonnyj@mirea.ru (N.B.); andreas.a.k@edu.mirea.ru (A.C.); klimov@mirea.ru (A.K.); lavrov_s@mirea.ru (S.L.); 2 Institute for Molecules and Materials, Radboud University, 6525 AJ Nijmegen, The Netherlands; dinar.khusyainov@ru.nl; 3 Univ. Lille, CNRS, Centrale Lille, Univ. Polytechnique Hauts-de-France, UMR 8520 -IEMN, 59000 Lille, France; nicolas.tiercelin@iemn.fr; 4 Prokhorov General Physics Institute of RAS, 119991 Moscow, Russia; vlpreobr@yandex.ru

**Keywords:** spintronic emitters, THz radiation, THz-TDS, ferromagnet, semiconductor

## Abstract

In this article, we investigate optically induced terahertz radiation in ferromagnetic FeCo layers of varying thickness on Si and SiO_2_ substrates. Efforts have been made to account for the influence of the substrate on the parameters of the THz radiation generated by the ferromagnetic FeCo film. The study reveals that the thickness of the ferromagnetic layer and the material of the substrate significantly affect the generation efficiency and spectral characteristics of the THz radiation. Our results also emphasize the importance of accounting for the reflection and transmission coefficients of the THz radiation when analyzing the generation process. The observed radiation features correlate with the magneto-dipole mechanism, triggered by the ultrafast demagnetization of the ferromagnetic material. This research contributes to a better understanding of THz radiation generation mechanisms in ferromagnetic films and may be useful for the further development of THz technology applications in the field of spintronics and other related areas. A key discovery of our study is the identification of a nonmonotonic relationship between the radiation amplitude and pump intensity for thin films on semiconductor substrates. This finding is particularly significant considering that thin films are predominantly used in spintronic emitters due to the characteristic absorption of THz radiation in metals.

## 1. Introduction

Advancements in the fields of nanomagnetism and spintronics have enabled the initial use of ultrafast optical pulses to generate terahertz radiation, leveraging the physics of ultrafast spins [1,2,3,4]. The primary physical mechanisms in ferromagnetic- [5] and ferromagnetic/nonmagnetic-type [6] structures are believed to be an ultrafast demagnetization and the inverse spin Hall effect (ISHE) [4]. Pulsed THz radiation emitters based on ferromagnet/metal structures are at the cutting edge of THz technology today. Currently, numerous studies and reviews discuss the efficiency of THz spintronic emitters, depending on the choice of material for a ferromagnet and a nonmagnetic conductor with substantial spin–orbit interaction [2,3].

Spintronic THz radiation sources are an intriguing subject of intensive research, as their efficiency is on par with known types of THz radiation sources, such as photoconductive antennas, nonlinear crystals, etc. [7,8,9]. A significant advantage of spintronic THz emitters is the development of new methods for controlling the THz polarization and spectrum using magnetic [9,10,11,12] and electric fields [13,14,15,16]. Consequently, a thorough understanding of the fundamental processes responsible for the THz signal formation has become an important task in THz spintronics research. More studies are emerging related to fundamental research aimed at explaining the causes of spin and charge effects [17,18,19,20,21].

Various methods influence the specified parameters by affecting the crystallographic properties of materials or the types of mechanisms. Interface effects on material properties are a well-known and crucial component in the study of spintronic THz sources. For instance, employing a semiconductor substrate for a magnetic layer has demonstrated the possibility of superdiffusive spin currents, which, in turn, allow the formation of spin currents with record spin polarization [22]. The effect of layers on semiconductor generation efficiency has been explored using monatomic transition metal dichalcogenide layers [22,23]. Utilizing different types of substrates can also serve as a means to control defect density, which subsequently enables the additional control of the spectral width by introducing defects into the sample. However, this results in a decrease in the peak field amplitude [24]. Overall, research in THz spintronics continues to grow, with increasing numbers of researchers investigating the fundamental aspects and practical applications of this technology. Uncovering the mechanisms that determine THz radiation generation in such materials is of utmost interest at present.

In the rapidly evolving field of spintronics, systematic investigations into the properties of different materials have proven to be invaluable. Our latest research presents a novel exploration of THz emission across a series of samples with varied thicknesses. This investigation is unique, as all samples were fabricated using the same technology, and were analyzed on both dielectric and semiconductor substrates, providing a comprehensive understanding of the effects of these variables on THz emission.

With this in mind, our research focused on a series of spintronic THz emitters, comprising ferromagnetic FeCo films of varying thicknesses deposited on distinct substrates, specifically Si and SiO_2_. The main goals of our study were to shed light on the terahertz amplitude generation mechanism and to investigate how the substrate affected the parameters of the emitted radiation. Our findings primarily enhance our understanding of THz radiation generation processes and consider additional factors contributing to the parameters of THz radiation, which ultimately broadens their potential applications across diverse fields.

This research not only enriches our understanding of the intricate dynamics between material thickness and substrate type in relation to THz emission but also provides critical knowledge that could drive advancements in the development of spintronic emitters. As we delve deeper into the intricate world of spintronics, insights such as these propel us closer to harnessing the full potential of this technology, opening up exciting possibilities for the future.

## 2. Materials and Methods

We utilized FeCo spintronic emitters of varying thicknesses (5 nm, 10 nm, 20 nm, and 40 nm) and produced them using a radiofrequency (RF) diode sputtering on a LEYBOLD Z550 system. We maintained a standard base vacuum in the deposition chamber at approximately 3 × 10^−7^ mbar to ensure a clean environment. During the deposition process, we positioned the substrates within an in-plane field with an estimated strength of 80 kA/m. This technique imprinted an in-plane magnetic anisotropy on the layers, enhancing their magnetic characteristics. For the sputtering procedure, we employed circular targets of 4 inches in diameter. The process was conducted at an RF power of 440 W, under a pure argon atmosphere, with a pressure of 2 × 10^−3^ mbar. In order to achieve the required precision in layer thicknesses, we implemented a deposition approach using a rotary turntable substrate holder. This holder oscillated at a calibrated speed, ensuring a uniform and precise deposition of the layers.

**Figure 1:**
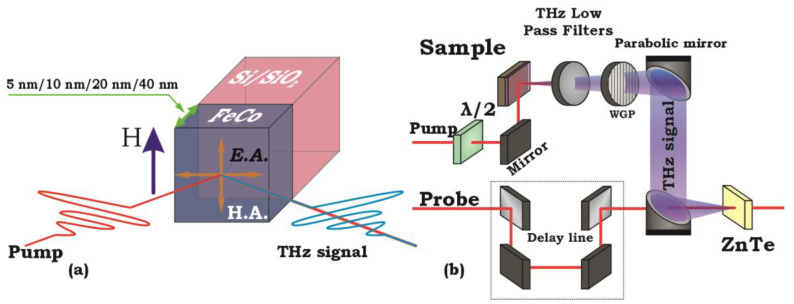
(**a**) A series of samples with FeCo layer thicknesses of 5 nm, 10 nm, 20 nm, and 40 nm deposited on Si and SiO_2_ substrates. The samples were grown under a constant magnetic field to form a uniaxial magnetic anisotropy. Arrows indicate the directions of the hard and easy axes formed in the film. (**b**) Schematic of the THz-TDS setup.

The sample structure is illustrated in Figure 1a. To achieve uniaxial magnetic anisotropy, the FeCo ferromagnetic layer was deposited under a constant magnetic field. During the deposition process, the sample was mounted on a nonmagnetic holder between two magnetic poles, allowing for magnetic fields up to approximately 30 mT to be applied within the sample plane [25]. The formation directions of the hard (H.A.) and easy magnetization axes (E.A.) are indicated by arrows in Figure 1a.

The study was carried out by the method of terahertz time-domain spectroscopy (THz-TDS) in reflection geometry (Figure 1b). A femtosecond laser system with a regenerative amplifier (Avesta) with a pulse duration of 35 fs, repetition rate of 3 kHz, and a wavelength of 800 nm was used as a source of laser radiation. The maximum energy density in a pump pulse was ~1 mJ/cm^2^. Upon leaving the regenerative amplifier, the laser beam was split into two distinct components, the pump and the probe beams, maintaining a proportion of 9:1, respectively. The pump beam underwent modulation through an optomechanical chopper before being directed towards the experimental sample, which was strategically situated in a magnetic field between the cores of an electromagnet. Portions of the pump beam not absorbed by the spintronic emitter were trimmed off using a terahertz filter. The resulting useful THz signal was initially collected and collimated, then refocused onto a zinc telluride crystal by a pair of parabolic mirrors. The polarization of the produced THz pulse was monitored by a wire-grid polarizer (WGP) [11]. To synchronize the arrival time of the pump and probe pulses, an optical delay line was employed, consisting of a computer-guided mobile platform housing a retroreflector. The probe beam was subsequently focused onto a nonlinear ZnTe optical crystal. The powerful field of the terahertz pulse invoked a nonlinear electro-optical effect within the crystal, causing a rotation of the probe beam’s linear polarization due to birefringence. The probe beam then proceeded through the analyzer, a Glan–Taylor prism strategically aligned with the polarizer axis, and its signal was picked up by a photodiode. The power and polarization of all the beams were regulated using a half-wave plate and a Glan–Taylor prism. During the entirety of the experiment, the pump and probe beam maintained a linear polarization in the plane of the table (P-polarization).

A zero-point electro-optical regime was used for the Δ*S(t)* signal proportional THz detection [26]. In order to increase the signal-to-noise ratio, the intensity of the reflected probe signal recorded by a synchronous detector (lock-in amplifier SR830) was given by: Δ𝑆(𝑡) = 𝐸_THz_ ∗ 𝐶𝑜𝑠(𝛩(𝑡)), where 𝛩 is the phase difference between Δ𝑆(𝑡) and the reference signal, t is the delay time [27], 𝐸*_THz_* is a signal recorded by a photodiode and dependent on the magnitude and polarization of the THz field [28]. The sample was positioned between the cores of the magnet, a configuration that facilitated the acquisition of THz hysteresis loops. Additionally, the sample was mounted on a rotating slide, allowing us to gather hysteresis loops corresponding to various orientations of the heavy and light axes of magnetization.

In our experiment, three types of measurements were conducted for all samples: (1) the dependence of the THz time-domain waveform on the pump fluence; (2) the dependence of the THz time-domain waveform on the magnetic field; (3) the dependence of the maximum THz signal on the magnetic field.

## 3. Results

**Figure 2:**
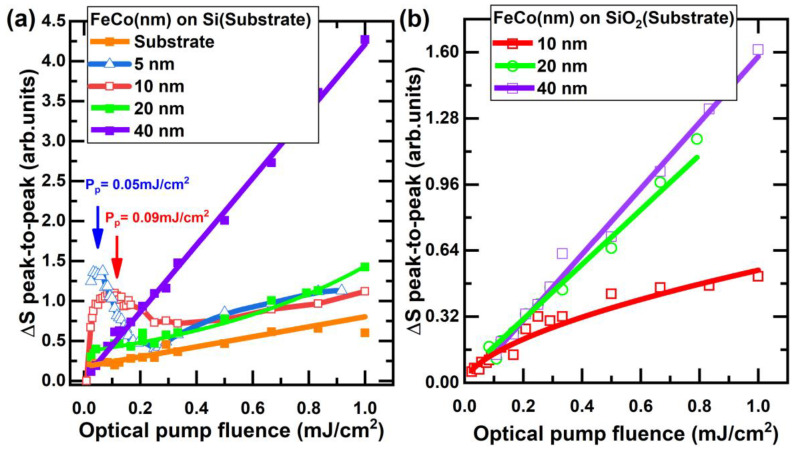
Dependence of THz signal on optical pump fluence for (**a**) Si and (**b**) SiO_2_ substrates. The scales on the vertical axes of figures (**a**,**b**) are comparable.

Figure 2 shows the results of the measurement of the THz signal dependence on the optical pump fluence. Figure 2a shows the peak-to-peak amplitude for a series of spintronic emitters on a silicon substrate. A nonlinear dependence was found on a spintronic emitter with FeCo layers of thickness 5 nm and 10 nm. Extremums for 5 nm and 10 nm thick FeCo samples were observed at pump fluences of 0.05 mJ/cm^2^ and 0.09 mJ/cm^2^, respectively. A strong nonlinearity was observed at a low fluence of up to 0.4 mJ/cm^2^. In the thicker samples, a linear dependence typical for such emitters was observed. The nature of the extremums is discussed below. The generation amplitude of the THz radiation for a FeCo film 5 nm thick did not depend on the magnetic field, neither in magnitude nor in phase (Figure S1).

Figure 2b provides a comprehensive visual depiction of the THz signal with respect to the optical pump fluence. This fluence varied from 0.01 mJ/cm^2^ to 1 mJ/cm^2^ for thin-film FeCo samples, all of which were placed on a SiO_2_ substrate. A striking linear correlation was evident for FeCo films of 40 nm and 20 nm thickness when observed in relation to the SiO_2_ substrate. However, such linearity was conspicuously absent for the 10 nm film.

An intriguing point to note is the absence of a data point for the optical pump fluence of 1 mJ/cm^2^ for the 20 nm thick film. This gap in the data set can be attributed to an unexpected degradation of the sample, potentially induced by a significant spike in optical power. Despite these missing data, the overall linear trend between the THz signal and optical pump fluence inferred that the absence of the data point for the 20 nm film at an optical pump fluence of 1 mJ/cm^2^ would likely not affect the overall interpretation or yield any new insights.

**Figure 3:**
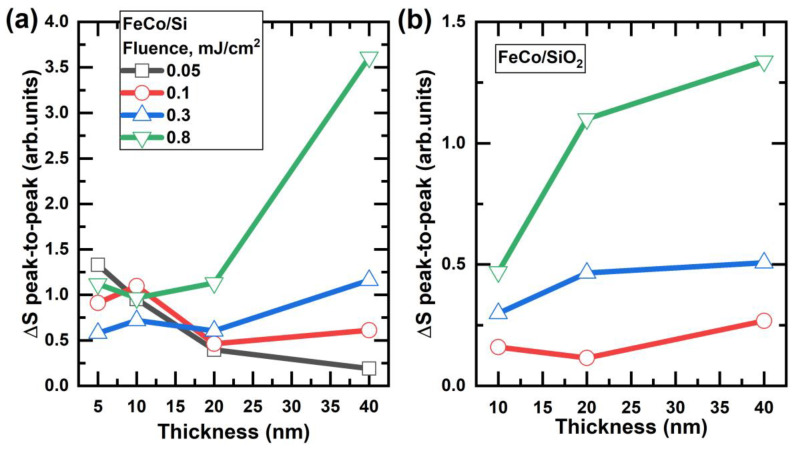
Relationship between THz radiation amplitude and FeCo layer thickness for (**a**) Si and (**b**) SiO_2_ substrates at various energy densities of the pump laser beam. The vertical axes’ scales in figures (**a**,**b**) are comparable.

The impact of the substrate on the THz signal was also apparent in the varying rates of emission amplitude increase with the escalation of the pumping fluence for 40 nm-thick samples deposited on Si and SiO_2_ substrates. This suggests that the choice of substrate material can have a significant impact on the performance of THz devices. The data shown in Figure 3 provide a visual representation of the relationship between the FeCo film thickness and THz signal for different substrate materials.

Additionally, at relatively high fluence, there appeared to be a similar trend in both substrates, where increasing the film thickness resulted in a higher amplitude. This can be explained by the fact that increasing the thickness leads to a higher net magnetization of the bulk sample, which contributes to the generation of THz radiation. Due to the low amplitude of the THz signal, there were no data for 0.05 mJ/cm^2^ on SiO_2_.

**Figure 4:**
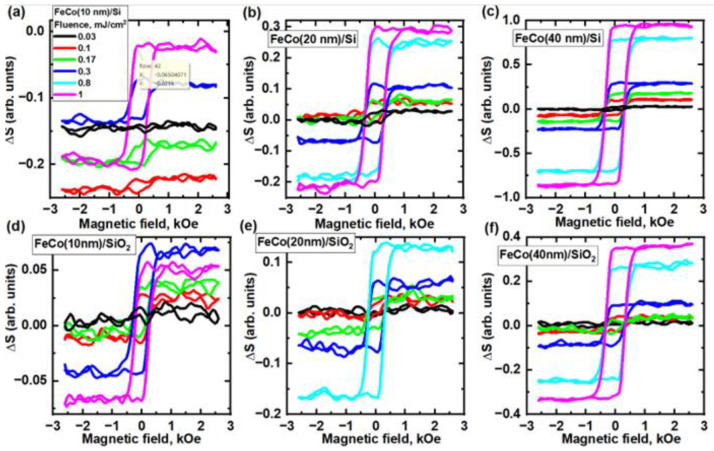
Dependence of the THz signal on the magnetic field for (**a**) 10 nm, (**b**) 20 nm, and (**c**) 40 nm thick FeCo films on Si substrates. Dependence of the THz signal on the magnetic field for (**d**) 10 nm, (**e**) 20 nm, (**f**) 40 nm thick on SiO_2_ substrates.

To verify the magnetic origin of THz generation, THz hysteresis loops were measured (Figure 4). As observed, these measurements correlated with the fluence dependencies shown in Figure 2. Furthermore, they provided crucial insights into the laser low-fluence region. Interestingly, the nonlinear dependencies observed were nonmagnetic in nature, which was evidenced by the absence of any signal dependency on the magnetic field in low-fluence measurements. Notably, these effects were only observed in thin samples on a Si substrate. We hypothesize that this effect stems from nonlinear features of an optically induced electron transfer through the metal/semiconductor interface. The details deserve special consideration outside the scope of this work.

**Figure 5:**
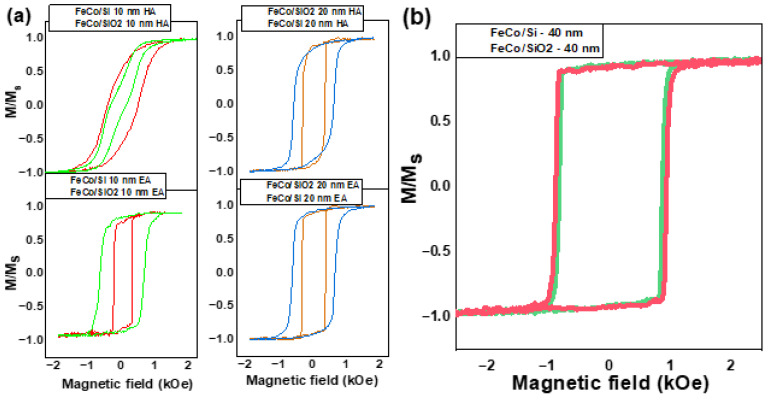
Transverse MOKE hysteresis loops for (**a**) 10 nm and 20 nm thick films for a magnetic field directed along E.A. and H.A. and (**b**) a 40 nm thick film for a magnetic field directed along E.A. on SiO_2_ and Si substrates.

This research presents the results of hysteresis loop analysis conducted on three FeCo film samples with varying thicknesses: 10 nm, 20 nm, and 40 nm. The FeCo films, deposited on both silicon (Si) and silicon dioxide (SiO_2_) substrates, were analyzed in detail using a setup featuring the transverse magneto-optical Kerr effect (transverse MOKE). This allowed us to conduct a comprehensive examination of their magnetization properties. The collected data are presented in Figure 5.

Upon comparing the hysteresis loops, a noticeable trend emerged. As the FeCo film thickness increased, the “E.A.”-type anisotropy, prominent in the 10 nm sample, became less pronounced in the 20 nm sample and was virtually absent in the 40 nm film. The coercive force (Hc) in the “E.A.” direction was measured for each sample. The 10 nm sample displayed a coercive force of 290 Oe on the Si substrate and 670 Oe on the SiO_2_ substrate. The saturation field (Hs) in the “H.A/” direction was similar for both substrates, measuring at approximately 1600 Oe. The 20 nm samples demonstrated a coercive force of about 320 Oe on the SiO_2_ substrate and roughly 640 Oe on the Si substrate, in both magnetization directions. The 40 nm samples exhibited coercive forces of 830 Oe and 920 Oe on the Si and SiO_2_ substrates, respectively.

The data suggested that as the thickness of the FeCo film increased, the “E.A.” type anisotropy decreased. Additionally, the coercive force (Hc) exhibited a proportional shift in samples deposited on Si substrates, while a nonmonotonic shift was observed in samples on SiO_2_ substrates. The results of this study highlight the significant influence of the substrate on the magnetic properties of FeCo film samples with thicknesses up to 20 nm. This is evident in the distinct alterations observed in the shape of the hysteresis loop depending on the type of substrate, as shown in Figure 5a. These modifications in the loop’s shape, attributed to the substrate, were substantial as long as the sample’s thickness did not exceed 20 nm. Notably, when the FeCo film sample’s thickness increased to 40 nm, no changes in the parameters of the magnetic hysteresis were detected, as shown in Figure 5b. These observations emphasize the complex relationship between the thickness of the FeCo film and the substrate type on its magnetic properties.

## 4. Model

To further investigate our observations, we utilized a model inspired by the research outlined in study [29]. The authors of that study proposed a correlation between the relaxation time of the spin subsystem and the generation of the terahertz effect. Essentially, a longer duration of energy transfer from electrons to spins and lattice resulted in a more pronounced terahertz signal.

The conventional two-temperature model illustrates the energy distribution between the lattice and electron subsystems following optical excitation. The process begins with a sharp rise in electron temperature due to their lower heat capacity compared to the lattice. Once the optical pulse ceases, the energy transfers to the lattice, gradually increasing its temperature until a state of equilibrium is achieved between the electrons and the lattice. However, this model does not consider a third crucial subsystem—the spins. An optical pulse creates a local nonequilibrium distribution of spins [30], a factor of critical importance in the creation of THz radiation. This local spin gradient is a key player in THz generation as it is the source of both ultrafast demagnetization and ISHE mechanisms.

The mechanism through which this subsystem relaxes determines which process is accountable for the THz emission. The relaxation mechanism of this subsystem determines the source of the THz emission, whether it stems from the ISHE—characterized by the transition of spins into charge currents in the metal—or ultrafast demagnetization, which involves the transition from a nonequilibrium spin distribution to an equilibrium state. Therefore, to fully comprehend the origin of THz radiation, it is crucial to consider both energy transfers, from electrons to lattice and from electrons to spins.

Here are the simple equations that were used to describe our results. The electric field generated by the emitter according to the magneto-dipole mechanism is described by the following equation [5]:

(1) $$E_{y}\left(t\right)=\frac{\mu_{0}}{4\pi^{2}r}\frac{\partial^{2}M_{x}}{{\partial t}^{2}}\left(t-\frac{r}{c}\right),$$

where $\mu_{0}$ is the vacuum magnetic permeability, $\frac{\partial^{2}M_{x}}{{\partial t}^{2}}$ is the second derivative of the *M_x_* magnetization component, and *r* is the distance to the magnetic dipole. The changing magnetization is described by a two-exponential phenomenological dependence [29]:

(2) $$\partial_{t}M(t)\propto \Theta (t)[A_{es}\exp\left(-\Gamma_{es}t\right)-A_{ep}\exp\left(-\Gamma_{ep}t\right)]⨂G(t),$$

where $A_{es}=\frac{{(\Gamma }_{es}-R\Gamma_{ep})}{{(\Gamma }_{es}-\Gamma_{ep})}$ and $A_{ep}=\frac{(1-R)\Gamma_{ep}}{{(\Gamma }_{es}-\Gamma_{ep})}$ are the amplitudes of the electron-spin and electron-phonon relaxation, respectively, $\Gamma_{ep}$ and $\Gamma_{es}$ are the correspondent time constants, Θ(*t*) is the Heaviside function, and *G(t)* is the Gaussian function.

**Figure 6:**
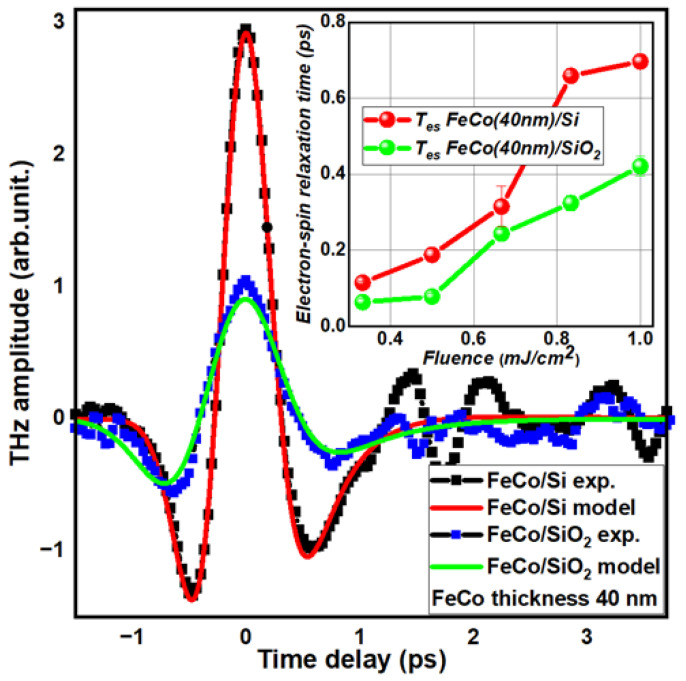
Example of fitting with the described model for 40 nm FeCo on a Si substrate. Electron-spin relaxation times extracted from approximation depicted in the inset (the line is a guide to the eye).

The experimental fluence dependences for different thicknesses of FeCo films on different substrates were approximated by the obtained final expression, and the electron-spin relaxation times were extracted from them (Figure 6).

Here, we can see that this model accurately describes our experimental results using only three parameters: the electron-phonon relaxation time, the electron-spin relaxation time, and the time delay shift which corresponds to fixing the uncertainty in choosing a zero point in the experimental data when converting from steps of delay line from micrometers to actual time delay. The electron-spin relaxation time was found to be dependent on the laser fluence. The dependence was almost linear within the order of magnitude. For the 40 nm FeCo sample on a Si substrate under a laser fluence of 1 mJ/cm^2^, we determined $\tau_{es}$ = 650 fs. For all other samples, the values of $\tau_{es}$ were located between 100 and 400 fs. The $\Gamma_{ep}$ values were larger than 1 ps, as expected based on previous work [31].

## 5. Discussion

Upon analyzing the samples using the transverse MOKE method (Figure 5a), we confirmed that the substrate influenced the magnetic properties of the films. This was due to the substrate’s contribution to the FeCo film’s crystallographic properties during deposition. Consequently, for films with thicknesses of 10 nm and 20 nm, the coercive field value, the saturation field for the H.A., and the magnetic loop’s nature changed. We attribute these changes in the THz signal for the 10 nm and 20 nm film samples solely to the substrate’s crystallographic contribution, without associating them with any interface effects that might impact the generation mechanism of THz radiation, such as the demagnetization value.

**Figure 7:**
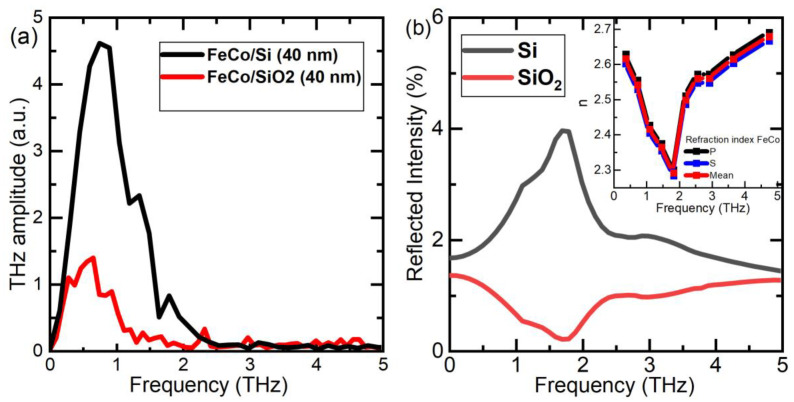
(**a**) THz signal frequency spectrum obtained from experimental data; (**b**) Calculated reflection values from FeCo/Si and FeCo/SiO_2_ interfaces. Inset: calculated refractive index for an FeCo layer on substrate Si and SiO_2_.

Interestingly, no crystallographic contribution from the substrate was observed in samples with a 40 nm FeCo thickness (Figure 5b). From the perspective of spintronic THz generation parameters in a magnetic film, the films on Si and SiO_2_ substrates were virtually identical. However, the terahertz amplitude and electron spin relaxation times derived from the two-temperature model of ultrafast demagnetization differed by more than 2.5 times (Figure 7a). With identical magnetic properties, the spin current should demagnetize in the same manner and exhibit the same relaxation time for their subsystems. Yet, we observed a difference in the T_es_ times (Figure 6 Inset) for the same incident optical fluence. In this case, we presumed the substrate’s influence. However, the substantial 40 nm film thickness precluded the impact of the metal/semiconductor interface [32].

To explain this discrepancy, we must first consider the significant difference in the refractive indices (and the reflection percentage) of the substrates within the terahertz wavelength range. For this purpose, we evaluated the reflection coefficients of the terahertz radiation at the FeCo/Si and FeCo/SiO_2_ interfaces. However, for thin FeCo films, the refractive index values in the terahertz range have not been defined. Thus, relying on the experimentally obtained frequency spectrum of the THz signal (Figure 7a), which was acquired using an FFT, we were able to estimate the refractive index values for FeCo. The reflection coefficients, reconstructed in this manner for the FeCo/Si and FeCo/SiO_2_ interfaces, are presented in Figure 7b, along with the corresponding refractive index values in the inset. As anticipated, we discovered that the reflection of Si was more than twice that of SiO_2_ (Figure 7b). It is worth noting that due to the high optical absorption of the pump radiation in the 40 nm thick FeCo film, the pump radiation did not pass through it and was absorbed only in the near-surface layer. Therefore, in this case, the pump was not sensitive to the choice of substrate.

## 6. Conclusions

In conclusion, the study analyzed FeCo THz spintronic emitters of varying thicknesses and substrates using the pump-probe technique for Si and SiO_2_ substrates and different thicknesses of FeCo film. The most efficient emitter in terms of THz signal was found to be a 40 nm thick FeCo film on a Si substrate. The study aimed to confirm that the sole mechanism of generation in such a structure was ultrafast demagnetization, as no other mechanisms were detected. Despite unusual nonlinear fluence dependencies in thin samples (5 and 10 nm), there was no dependency on the magnetic field, leading to the conclusion that these effects were merely interfacial. To further confirm the ultrafast demagnetization origin of the THz signal, a two-temperature model was utilized, which showed a strong agreement with experimental data. The findings of this research are vital for advancing high-performance spintronic emitters, showing that an increased THz signal can be achieved not only by modifying the magnetic properties of the structure but also by altering substrates. Combining these approaches could contribute to the development of even more efficient emitters.

## Data Availability

The data presented in this study are available on request from the corresponding author.

## Supplementary Materials

The following supporting information can be downloaded at: https://www.mdpi.com/article/10.3390/nano13111710/s1, Figure S1: Dependence of the THz signal on time delay for sample of FeCo 5 nm on Si substrate for 3 pump fluences (a) 0.025 mJ/cm^2^, (b) 0.25 mJ/cm^2^, (c) 0.9 mJ/cm^2^; Figure S2. Time domain waveform of the generated THz pulses from 40 nm FeCo films on Si (a,b) and SiO_2_ (c,d) for 2 power fluences 0.17 and 1 mJ/cm^2^; Figure S3. THz time-domain waveform and Fast Fourier Transform spectrum for FeCo thin films grown on Si substrate for 3 fluences 0.1, 0.3 and 0.8 mJ/cm^2^; Figure S4. Fast Fourier Transform spectrum for FeCo thin films grown on Si substrate for fluences range 0.03–1 mJ/cm^2^; Figure S5. THz time-domain waveform and Fast Fourier Transform spectrum for FeCo thin films grown on SiO_2_ substrate for 3 fluences 0.1, 0.3 and 0.8 mJ/cm^2^; Figure S6. The Fast Fourier Transform spectrum for FeCo thin films, grown on SiO_2_ substrates, spans a fluence range of 0.03–1 mJ/cm^2^.
