# Supplementary material for: The Role of Ferromagnetic Layer Thickness and Substrate Material in Spintronic Emitters
Source: Nanomaterials (Basel). 2023 May 23;13(11):1710. doi: 10.3390/nano13111710 (PMC10254317; doi:10.3390/nano13111710)
Supplement: Supplementary file 1 [file nanomaterials-13-01710-s001.zip › nanomaterials-2390767-supplementary.pdf]

Supplementary Materials:

The Role of Ferromagnetic Layer Thickness and Substrate Material in Spintronic Emitters

Figure S1 illustrates the time dependence of the THz signal for a 5 nm thick FeCo film on a Si substrate. The generation mechanism of THz radiation is not magnetic, as the THz signal does not depend on the magnetic field in either magnitude or phase. The main characteristic is the nonmonotonic dependence of the signal on pump power. Specifically, a change in power results in a change in the duration of the THz pulse. This is clearly observed in the series of figures S1 (a), (b), and (c).

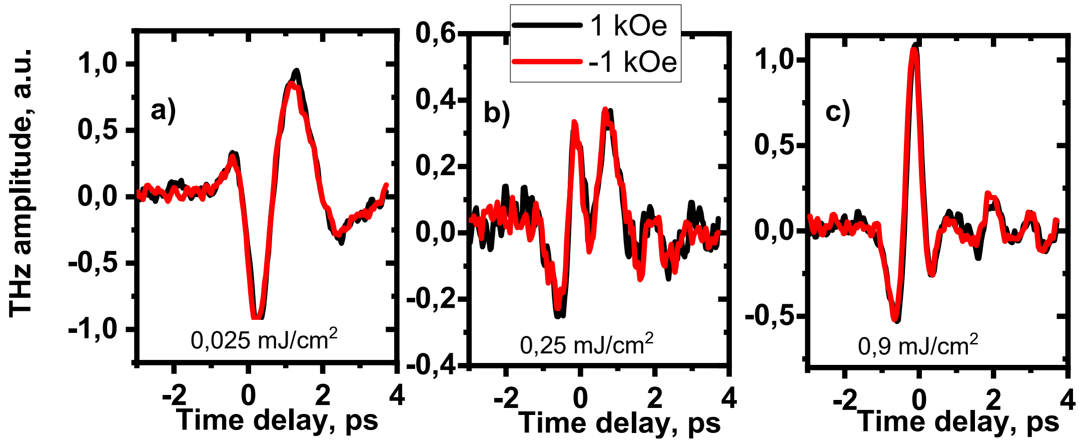

Fig. S1. Dependence of the THz signal on time delay for sample of FeCo 5nm on Si substrate for 3 pump fluences a) 0.025 mJ/cm^2, b) 0.25 mJ/cm^2, c) 0.9 mJ/cm^2.

Figure S2 displays the THz waveform for a 40 nm thick FeCo film. In a thick film (40 nm), the mechanism of THz generation is magnetic dipole. Demagnetization follows the superdiffusion scenario. The difference in THz amplitudes for Si and SiO2 is attributed to the variations in the optical properties of the substrates in the THz range; no role of spin transport in the semiconductor was identified.

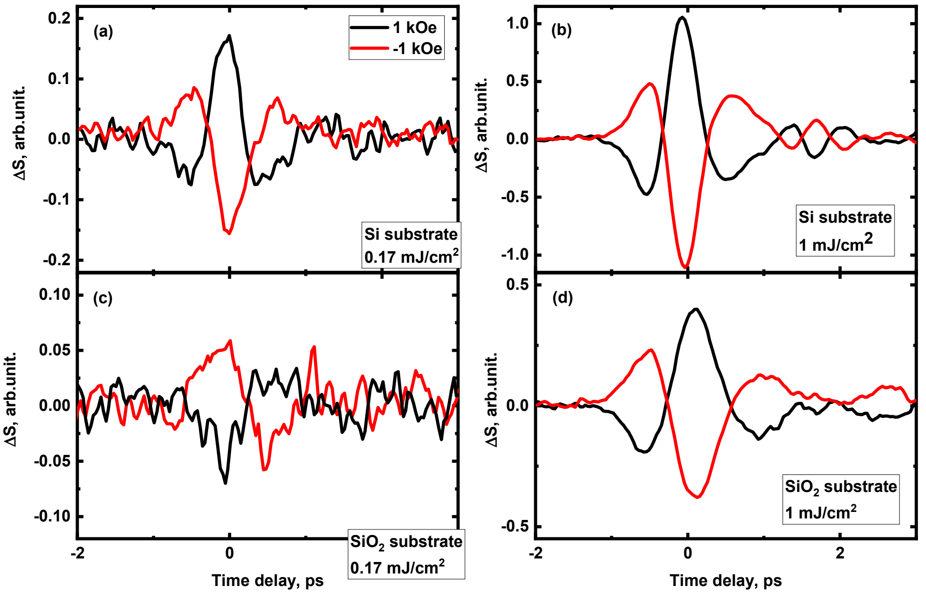

Fig. S2. Time domain waveform of the generated THz pulses from 40 nm FeCo films on Si a), b) and SiO_2 c), d) for 2 power fluences 0.17 and 1 mJ/cm^2

Figure S3 presents the time dependences of the THz signal for FeCo films with thicknesses of 5 nm, 10 nm, 20 nm, and 40 nm, deposited on a Si substrate. These time dependences were converted into a THz spectrum using the Fourier transform method (see

Figure S3 on the right). Results are displayed for three power levels: 0.1 mJ/cm^2 (upper S3), 0.3 mJ/cm2 (middle S3), and 0.8 mJ/cm2 (lower S3).

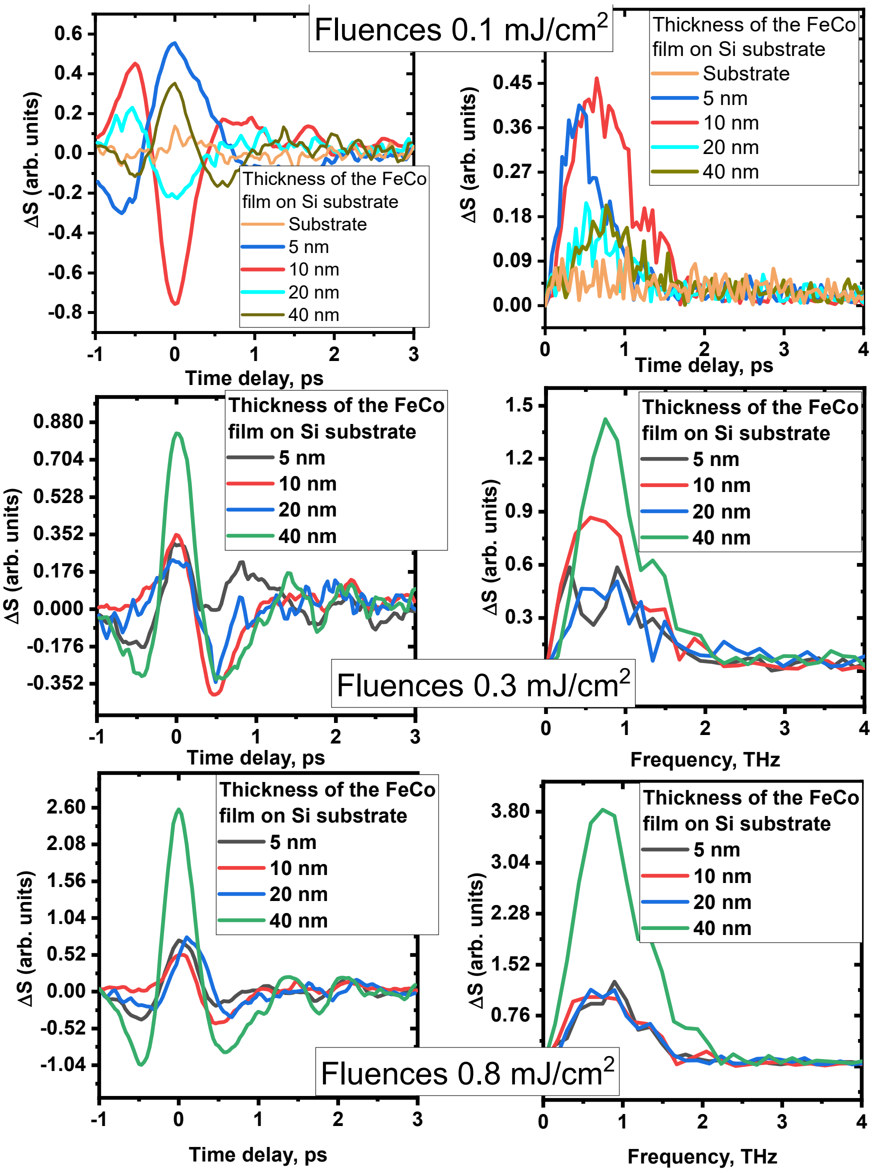

Fig. S3. THz time-domain waveform and Fast Fourier Transform spectrum for FeCo thin films grown on Si substrate for 3 fluences 0.1, 0.3 and 0.8 mJ/cm^2.

Figure S4 displays the results of normalizing the THz spectra for FeCo films of varying thicknesses, deposited on a silicon substrate, taking into account the THz signal. The results are presented for power densities ranging from 0.03 mJ/cm^2 to 1 mJ/cm^2. They clearly demonstrate a resonant frequency shift from approximately 0.5 THz to about 1 mJ for a 5 nm thick FeCo film. The same observation holds for the 10 nm thick FeCo film. Nonmonotonicity suggests the presence of competing processes. If pumping penetrates through 5 nm and 10 nm thick films into the semiconductor, and the work functions for both the metal and semiconductor are similar (about 4 eV), two opposing tendencies of electron transfer through the interface emerge: diffusion of hot electrons from the metal into the semiconductor, and transfer of photoelectrons from the semiconductor to the metal. With weak pumping, one effect may dominate, while with strong pumping, it can saturate and be suppressed by the other.

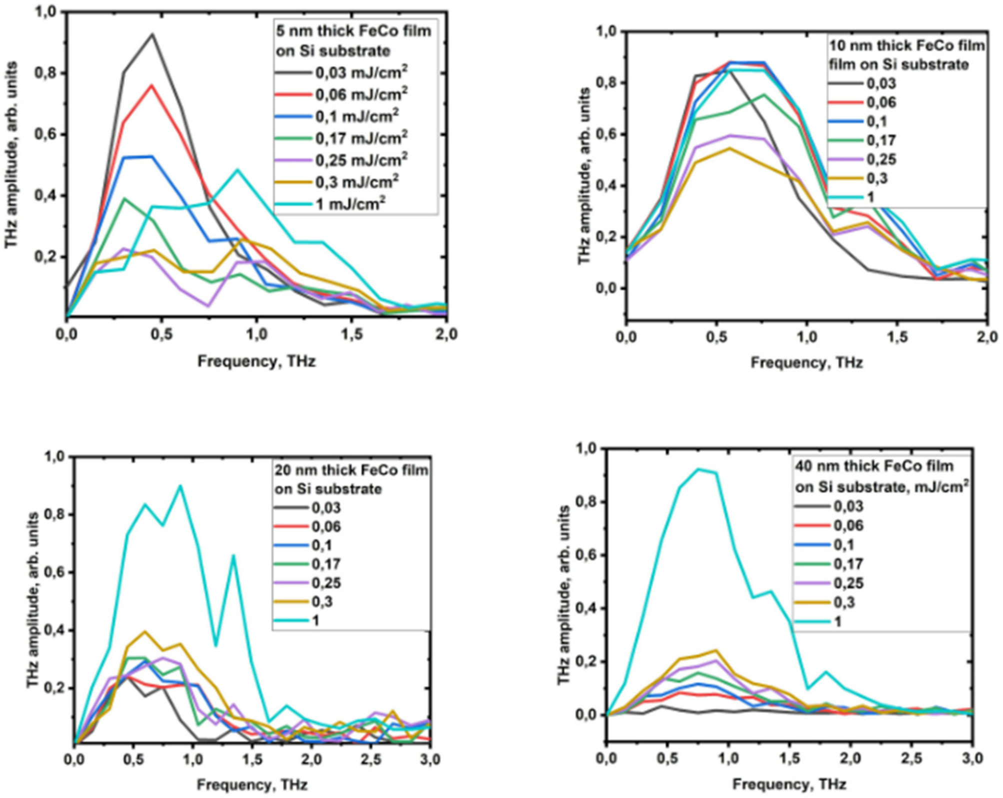

Fig. S4. Fast Fourier Transform spectrum for FeCo thin films grown on Si substrate for fluences range 0.03 – 1 mJ/cm^2.

Figure S5 presents the THz signal results obtained for FeCo films on a SiO_2 substrate. The possibility of saturation of diffusion currents is indirectly supported by the results for a 10 nm thick film on SiO_2. If we assume that the nonmagnetic nature of THz generation is due to currents through the interface, then nonmonotonicity of the THz signal amplitude is observed. In this context, it becomes clear why such an effect is not present for a thicker film, as the pumping does not penetrate into the semiconductor through it.

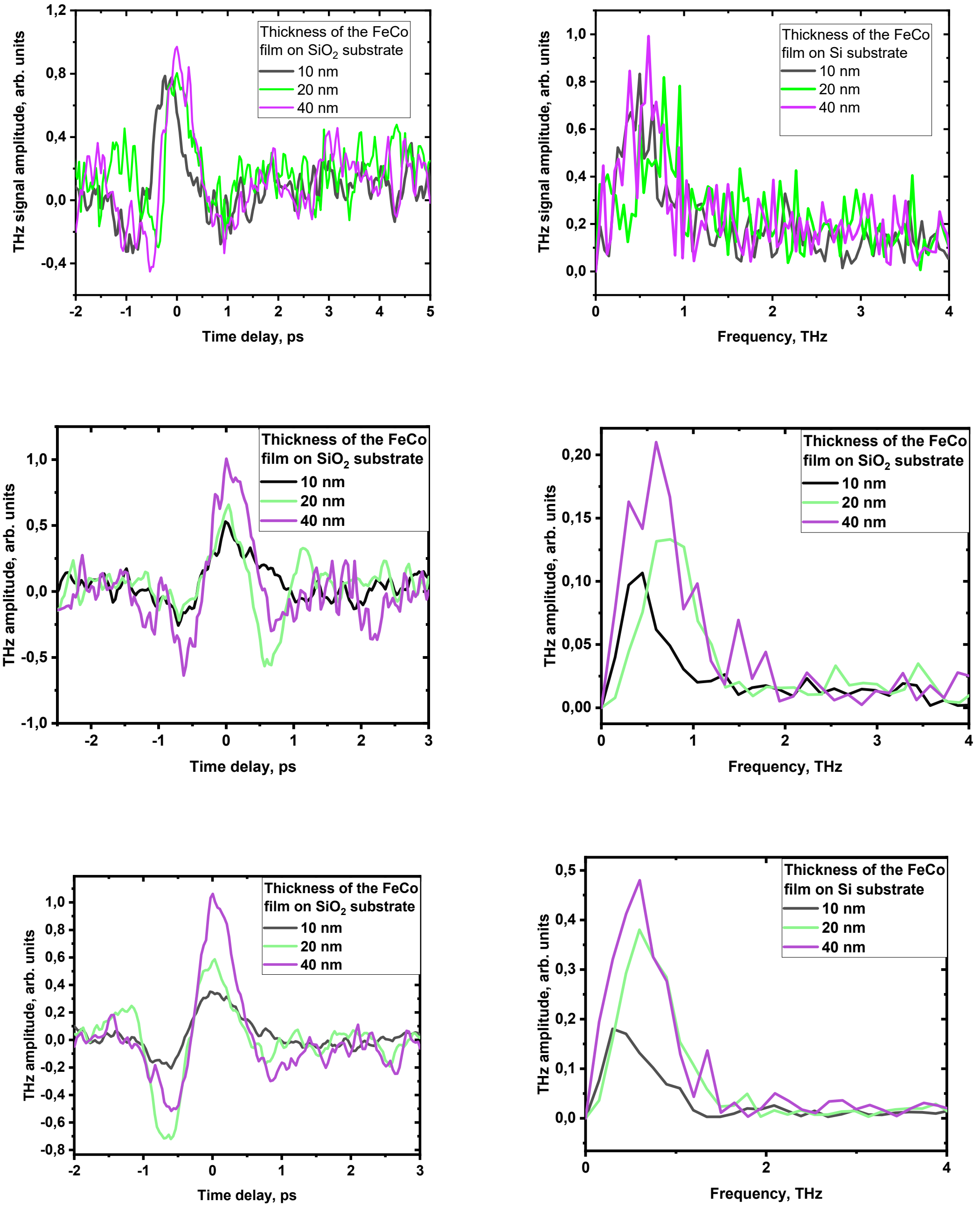

Fig. S5. THz time-domain waveform and Fast Fourier Transform spectrum for FeCo thin films grown on SiO_2 substrate for 3 fluences 0.1, 0.3 and 0.8 mJ/cm^2.

Fig. S6 displays the Fast Fourier Transform (FFT) spectrum for FeCo thin films grown on SiO_2 substrates, covering a fluence range from 0.03 to 1 mJ/cm^2. The spectrum illustrates the frequency response and characteristics of the THz signal generated by these films, providing insights into their behavior and properties across the specified fluence range.

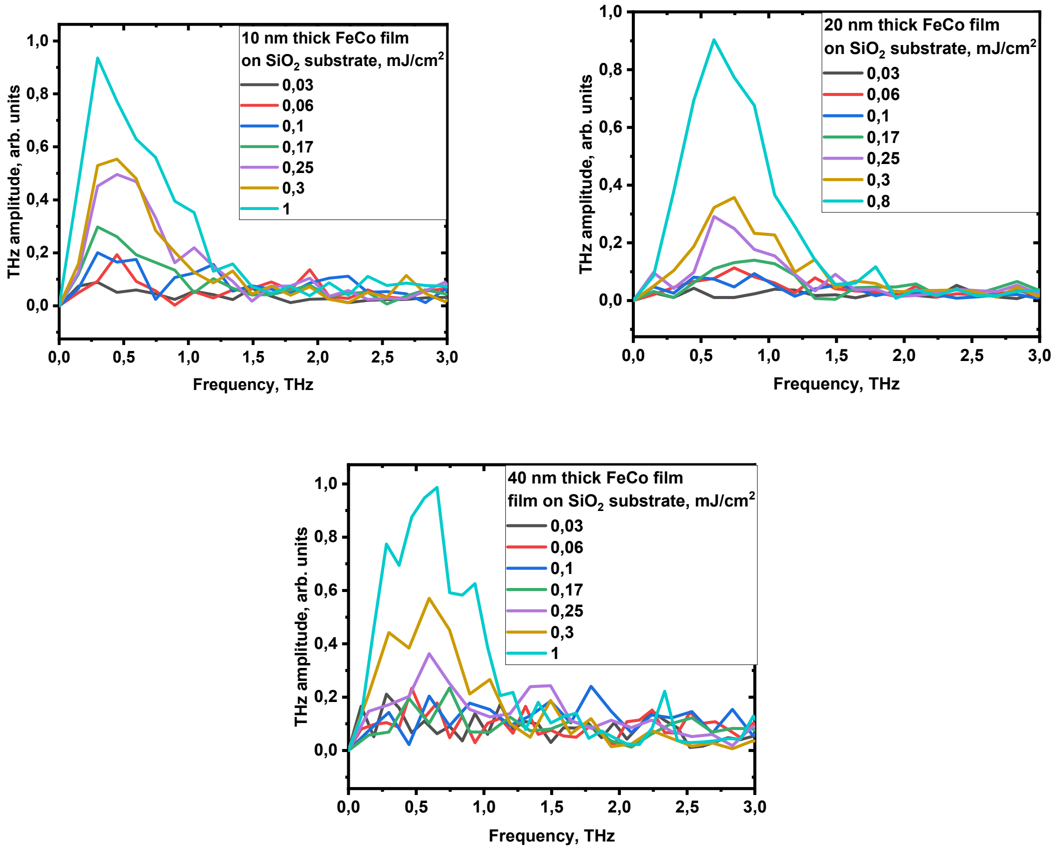

Fig. S6. The Fast Fourier Transform spectrum for FeCo thin films, grown on SiO_2 substrates, spans a fluence range of 0.03 – 1 mJ/cm^2.
